# Critical Steps of *Plasmodium falciparum* Ookinete Maturation

**DOI:** 10.3389/fmicb.2020.00269

**Published:** 2020-03-17

**Authors:** Giulia Siciliano, Giulia Costa, Pablo Suárez-Cortés, Angelo Valleriani, Pietro Alano, Elena A. Levashina

**Affiliations:** ^1^Dipartimento di Malattie Infettive, Istituto Superiore di Sanità, Rome, Italy; ^2^Vector Biology, Max Planck Institute for Infection Biology, Berlin, Germany; ^3^Department of Theory and Bio-Systems, Max Planck Institute of Colloids and Interfaces, Potsdam, Germany

**Keywords:** malaria parasite transmission, *Plasmodium falciparum*, *Anopheles coluzzii*, ookinete maturation, time course of zygote development, developmental bottleneck

## Abstract

The egress and fertilization of *Plasmodium* gametes and development of a motile ookinete are the first crucial steps that mediate the successful transmission of the malaria parasites from humans to the *Anopheles* vector. However, limited information exists about the cell biology and regulation of this process. Technical impediments in the establishment of *in vitro* conditions for ookinete maturation in *Plasmodium falciparum* and other human malaria parasites further constrain a detailed characterization of ookinete maturation. Here, using fluorescence microscopy and immunolabeling, we compared *P. falciparum* ookinete maturation in *Anopheles coluzzii* mosquitoes *in vivo* and in cell culture *in vitro*. Our results identified two critical steps in ookinete maturation that are regulated by distinct mosquito factors, thereby highlighting the role of the mosquito environment in the transmission efficiency of malaria parasites.

## Graphical Abstract

Ookinete development within the mosquito blood bolus is a critical step in malaria transmission. Using time-series approaches, we identified two limiting steps of *Plasmodium falciparum* ookinete development *in vitro*: (i) inhibition of transition from round forms (stage I) to knobs (stage II), likely caused by an inefficient fertilization, and (ii) arrest of transition between stages III and IV, associated with morphological abnormalities during protuberance elongation and enlargement. The abnormal protuberance at stage IV prevents further nuclear migration from the round body to the appendage.

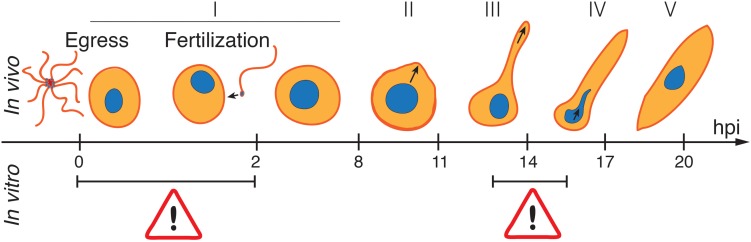

## Introduction

Malaria is a mosquito-borne disease, mainly caused by the protozoan parasite *Plasmodium falciparum*, which kills 429,000 people annually (World Health Organization [WHO], 2018). A key component of the global initiative to eliminate malaria is the transmission block of malaria parasites from humans to the mosquito vector. *Plasmodium* transmission is initiated within minutes after blood ingestion by the *Anopheles* mosquito. Within the mosquito midgut, the gametocytes transform into mature extracellular female and male gametes. The product of fertilization, the zygote, takes one day to transform into a motile ookinete, which then traverses the midgut epithelium to establish oocyst infection on the basal side. About 10–12 days later, each oocyst releases thousands of sporozoites that migrate to and invade the salivary glands, ready to be injected into a human at the next mosquito bite. During the first 24 h after blood meal, the parasites undergo a series of radical tightly regulated morphological changes: gamete roundup, egress and exflagellation, formation of a zygote, emergence of an elongated protuberance and transformation of a round zygote into a crescent-shaped motile ookinete. The early stages of gametocyte-to-gamete transformation are triggered by the mosquito-derived metabolite xanthurenic acid, which together with the temperature drop signals for gamete egress and exflagellation (Billker et al., 1998). Although fertilization and post-fertilization steps are critical for *Plasmodium* establishment in the mosquito, very little is known about molecular mechanisms and signals that orchestrate parasite sexual reproduction. Field studies suggested that pre-fertilization events do not reliably predict the outcome of mosquito infection, as the numbers of the blood-circulating gametocytes do not always correlate with the donor infectiousness to the insect vector (Bousema and Drakeley, 2011). Are post-fertilization events (the transition from zygote to ookinete) a more accurate proxy to forecast the efficiency of *Plasmodium* transmission?

To better characterize the post-fertilization events, several studies attempted to establish *P. falciparum* ookinete cultures *in vitro* (Carter et al., 1987; Warburg and Schneider, 1993; Bounkewa et al., 2010; Ghosh et al., 2010; Delves et al., 2017). While such cultures were successful for the rodent malaria parasite *Plasmodium berghei* (Janse et al., 1985; Sinden et al., 1985), the efficiency of *P. falciparum* conversion from round-shaped parasites to mature ookinetes is surprisingly low (0.45 to 16%) as compared to the estimated 50% efficiency *in vivo* (Carter et al., 1987; Bounkewa et al., 2010; Delves et al., 2017). The poor efficiency of *P. falciparum* gamete fertilization was identified as a major obstacle to efficient ookinete production *in vitro* (Ghosh et al., 2010; Delves et al., 2017). As the factors that promote fertilization *in vivo* remained obscure, a series of culture conditions (lipids, glucose, mosquito pupal extract, and red blood cell lysate additives, variations in hematocrit, pH, and gas; etc.) were tested to rescue the block in fertilization, albeit without success (Ghosh et al., 2010; Delves et al., 2017).

In spite of low efficiency, culturing *in vitro* provided a first classification of ookinete development based on morphology of Giemsa-stained cells (Ghosh et al., 2010). Besides the round-shaped zygote, five ookinete stages have been described: (I and II) retorts with a short protuberance attached to the round body; (III) retort with enlarged but not fully elongated protuberance; (IV) retorts with the thin protuberance at maximum elongation; (V) crescent or fluke-shaped mature ookinetes. The drawback of this classification was the unspecific basis of the Giemsa staining, which could not distinguish between residual non-activated gametocytes, developmentally blocked degenerated parasites, and mature ookinetes. Application of monoclonal antibodies that bound the zygote/ookinete surface antigen Pfs25 and/or the ookinete-specific intracellular enzyme chitinase helped the identification of mature ookinetes (Carter et al., 1987; Bounkewa et al., 2010; Ghosh et al., 2010; Delves et al., 2017). However, the antibodies were used only to count the mature ookinetes at a late time point or at the very beginning and very end of the process, thereby preventing a faithful reconstruction of the ookinete maturation stages.

Here, using live immunofluorescence microscopy, we report the temporal characterization of the zygote to ookinete development in the *P. falciparum*-infected *Anopheles coluzzii* mosquitoes and in cell culture *in vitro*. By comparing the time series of the zygote development *in vivo* and *in vitro*, we demonstrate a requirement of mosquito-derived factor(s) for gamete fertilization and identify a new critical stage of ookinete maturation.

## Materials and Methods

### Parasite Cultures

*Plasmodium falciparum* NF54 parasites were cultured in O^+^ human red blood cells (Haema, Berlin), at 37°C, in a 3% O_2_, 5% CO_2_, and 92% N_2_ atmosphere. Asexual cultures (0.5–5% of parasitemia) were maintained at 3–4% hematocrit in the complete medium composed of RPMI 1640 with L-glutamine and 25 mM HEPES, supplemented with 10% human A^+^ serum (Haema, Berlin), 10 mM hypoxanthine (c-c-Pro), and 20 μg/ml of gentamicin (Sigma). For gametocyte production, asynchronous asexual parasites were seeded at 1% parasitemia and 5% hematocrit, and complete medium without gentamicin was replaced daily for 15 days until mosquito infections. Stage V gametocytes were enumerated in a counting chamber (Neubauer) 14 days post seeding. On the same day, a sample from the culture was incubated for 15 min at room temperature in the presence of 20 μM  of xanthurenic acid (Sigma), and exflagellating clusters were enumerated in a counting chamber (Neubauer). The exflagellation rate was calculated as follows:

$$\frac{\mathrm{No}.\mathrm{of}\,\mathrm{exflagellating}\,\mathrm{clusters}/\mathrm{m1}}{\mathrm{No}.\mathrm{of}\,\mathrm{stage}\,V\,\mathrm{gametocytes}/\mathrm{m1}}$$

*P. falciparum* NF54 clone originated from Prof. Sauerwein’s laboratory, Radboud University Medical Center, Nijmegen, The Netherlands, and was authenticated for *Pfs47* by PCR on genomic DNA. *P. falciparum* asexual cultures were monthly tested for *Mycoplasma* contamination.

### Mosquito Rearing and Parasite Infections

*Anopheles coluzzii* (Ngousso S1 strain) was maintained at 29°C 70–80% humidity 12/12-h day/night cycle. For *P. falciparum* infections, mosquitoes were fed for 15 min on a membrane feeder with stage V gametocytes diluted in fresh red blood cells and human serum to 3.7 × 10^6^ stage V gametocytes/ml and 50% hematocrit. Infected mosquitoes were kept in a secured S3 laboratory at 26°C for up to 11 days according to national regulations (Landesamt für Gesundheit und Soziales, project number 297/13). Unfed mosquitoes were removed shortly after infection.

### Mosquito Dissections

For ookinete analyses, midguts of decapitated mosquitoes were dissected in 1 × phosphate-buffered saline (PBS) at the indicated time points (20 midguts per time point for each experimental condition). For oocyst counts, mosquitoes were killed in ethanol 70% at 11 dpi, midguts were dissected and incubated in 1% mercurochrome (Sigma) in water for 5–10 min. The oocyst numbers were enumerated under a light microscope Leica DM2000 LED.

### Ookinete Cultures *in vitro* and *ex vivo*

For *in vitro* experiments, ookinete cultures were set up by diluting gametocyte cultures 15 days post seeding in fresh complete medium to 5 × 10^6^ stage V gametocytes/ml, supplemented with 20 μM xanthurenic acid and incubated at 26°C in a siliconized tube until analyses.

For *ex vivo* experiments, midguts of infected mosquitoes were homogenized in complete medium containing 40 μg/ml of gentamicin and 5 μg/ml of Fungizone (Gibco) to decrease the microbial charge, incubated for 2 min at room temperature, washed (300 *g*, 4 min), resuspended in 500 μl of complete medium containing 40 μg/ml of gentamicin (Alpha Laboratories), and incubated at 26°C in a siliconized tube for an additional 21 h. Control *in vivo* samples were collected from mosquito bolus 23 h post infection (hpi), while control *in vitro* ookinete cultures were set up with the same gametocyte suspension prepared for mosquito infections, diluted 1:25 in complete medium without gentamicin and incubated at 26°C for 23 h. As mock control, one aliquot from *in vitro* cultures was subjected 2 h post seeding to the same gentamicin/Fungizone treatment used *ex vivo* and then cultured for an additional 21 h (Supplementary Figure 4).

### Ookinete Staining and Quantification

**FIGURE 1:**
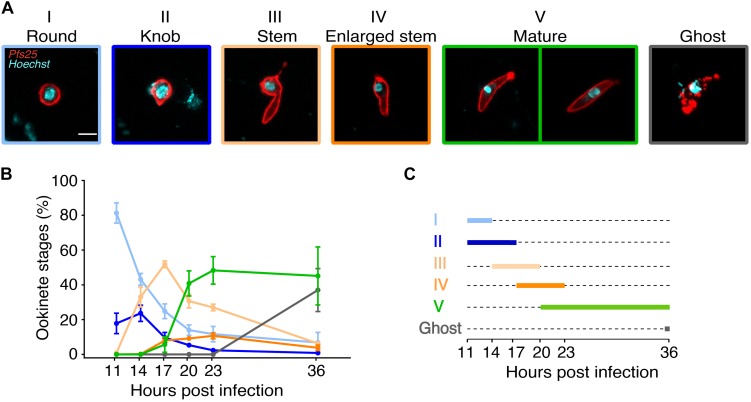
Kinetics of *Plasmodium falciparum* ookinete maturation in the mosquito bolus. *Anopheles coluzzii* mosquitoes were infected with *P. falciparum* gametocytes. Live parasites were isolated from a pool of 20 mosquito boluses and stained with anti-Pfs25 antibody at 11, 14, 17, 20, 23, and 36 h post infection (hpi). **(A)** Representative pictures of Pfs25-positive and Hoechst-stained ookinete stages as referred throughout this study (scale bar: 5 μm). Stage I: Round, indistinguishable from egressed female gamete. Stage II: Knob, small protuberance not exceeding in length the radius of the main spherical body. Stage III: Stem, elongated protuberance longer than the radius of the main spherical body. Stage IV: Enlarged stem, the elongated protuberance–still containing the nucleus–increasing in width. Stage V: Mature, terminally differentiated elongated form with or without residual spherical body; the nucleus has migrated from the residual spherical body to the stem. Ghost: elongated degenerated forms with non-continuous surface Pfs25 signal. **(B)** *In vivo* ookinete forms were classified as in **(A)**. Proportion of ookinete stages out of total Pfs25-positive parasites is indicated for each time point. Every point represents the mean ± SEM of three independent experiments (*N* = 3, *n* = 200 parasites counted per time point). **(C)** Schematic representation of the duration of ookinete stages in the mosquito vector described in **(A)**.

Hybridoma cells expressing anti-Pfs25 IgG (clone 4B7) were obtained from BEI Resources. IgG fraction was purified using Protein G Sepharose columns (GE Healthcare) and labeled to Alexa Fluor 568 (Life Technologies) in the Central Laboratory Facility (Deutsches Rheuma-Forschungszentrum, Berlin). Ookinetes were stained with anti-Pfs25-Alexa Fluor 568 antibody (7.5 μg/ml at 2 hpi and 1.5 μg/ml at 23 hpi) and Hoechst 1:500 (Molecular Probes) for 30 min at 4°C in PBS. Samples were washed (300 *g*, 4 min) and resuspended in PBS. The numbers of Pfs25-positive parasites were enumerated in a counting chamber (Neubauer) using a Leica DMRB fluorescence microscope and classified into stages using an Axio Observer Z1 fluorescence microscope equipped with an Apotome module (Zeiss) (Figure 1).

Ookinete conversion was defined as follows:

$$\frac{\mathrm{No}.\mathrm{of}\,\mathrm{converted}\,\mathrm{ookinetes}\,\left(\mathrm{Stage}\,\mathrm{II}\,\mathrm{to}\,\mathrm{IV}\right)}{\mathrm{No}.\mathrm{of}\,\mathrm{Pfs25}\,\text{-}\,\mathrm{positive}\,\mathrm{parasites}}$$

The proportion of mature ookinete was defined as follows:

$$\frac{\mathrm{No}.\mathrm{of}\,\mathrm{mature}\,\mathrm{ookinetes}\,\left(\mathrm{Stage}\,V\right)}{\mathrm{No}.\mathrm{of}\,\mathrm{Pfs25}\,\text{-}\,\mathrm{positive}\,\mathrm{parasites}}$$

Representative pictures were acquired with the Apotome module, and images were deconvoluted and corrected for phase contrast and local bleaching using ZEN 2012 software (Zeiss).

### Statistical Analysis

**FIGURE 2:**
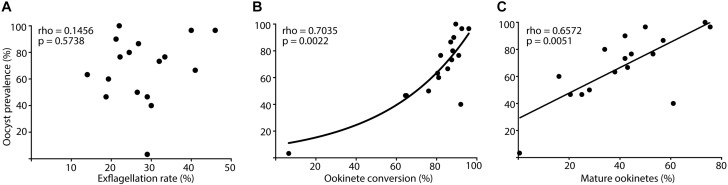
Correlation between male gamete activation and ookinete development rates with oocyst prevalence. *Anopheles coluzzii* mosquitoes were fed with *Plasmodium falciparum* gametocytes. Live parasites were isolated from the mosquito bolus and stained with anti-Pfs25 antibody at 24 h post infection (hpi), while oocysts were stained with mercurochrome and counted at 11 days post infection (dpi). Correlation between oocyst prevalence and **(A)** exflagellation rate (exflagellation clusters ml^–1^/gametocyte stage V ml^–1^) of gametocyte cultures 24 h before infection, **(B)** percentage of ookinete conversion (stages II to V ookinetes/total Pfs25-positive parasites), and **(C)** percentage of mature ookinetes (mature stage V ookinetes/total Pfs25-positive parasites). Every dot represents one independent experiment (*N* = 17), Pfs25-positive parasites (*n* = 200) were counted in pooled samples of 20 blood boluses, and oocyst prevalence was analyzed in dissected midguts (*n* = 30) per experiment. Rho- and *p*-values (Spearman correlation) are indicated on the top left corner of every graph, and lines show data trends for significant correlations **(B)**, exponential growth curve; **(C)**, linear regression.

No samples were excluded from the analyses. Mosquitoes from the same batches were randomly allocated to the experimental groups (age range: 1–2 days). The experimenters were not blinded to the group allocation during the experiment and/or when assessing the outcome. Statistical analysis for Figure 2 was performed with GraphPad Prism 8, and *p*-values below 0.05 were considered significant (^∗^*p* < 0.05) as indicated in the figures. The specific tests used are indicated in the figure legend.

**FIGURE 3:**
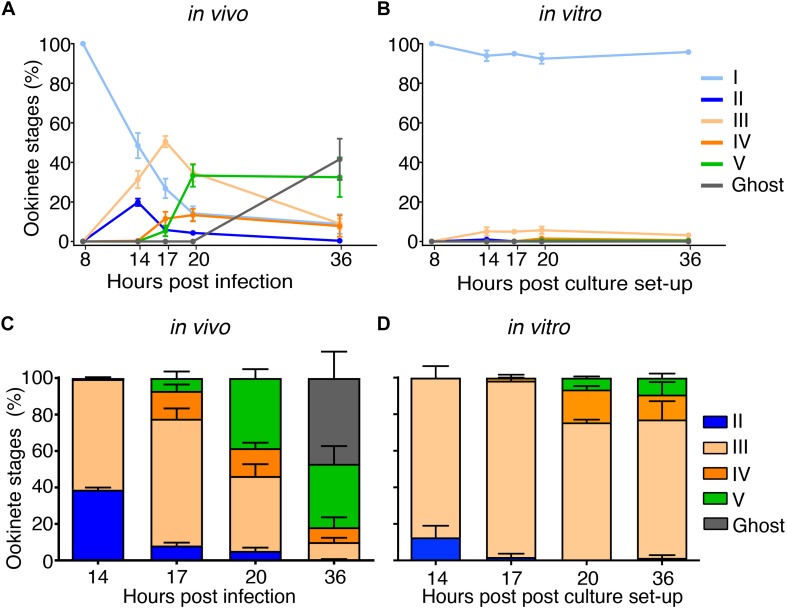
Parallel time course highlights two limiting steps in *in vitro* ookinete development. The same preparation of *Plasmodium falciparum* gametocytes was diluted and fed to mosquitoes or induced to undergo gametogenesis in a parallel time course of *in vivo* versus *in vitro* development. Parasites were isolated from a pool of 20 mosquito boluses or collected from the *in vitro* culture and stained with anti-Pfs25 antibody at 8, 14, 17, 20, and 36 h after feeding or culture setup. Ookinete forms were classified as in Figure 2A, and the proportions of ookinete stages on total Pfs25-positive parasites *in vivo* **(A)** or *in vitro* **(B)** are indicated for each time point. Every point represents the mean ± SEM of three independent experiments (**A:** *n* = 200, **B:** *n* = 500 parasites counted per time point). Relative proportions of ookinete stages out of total Pfs25-posititve parasites excluding round forms (stages II to V and ghosts) per each time point are shown *in vivo* **(C)** and *in vitro* **(D)**. Bar plots represent the mean proportions ± SEM of three independent experiments **(C)**, *n* = 309, 439, 515, and 449 and **(D)**, *n* = 91, 76, 114, and 63 Pfs25-positive parasites were analyzed per time point. The *z*-test was used to compare the mean proportion of each ookinete stage and its variances *in vivo* versus *in vitro*.

**FIGURE 4:**
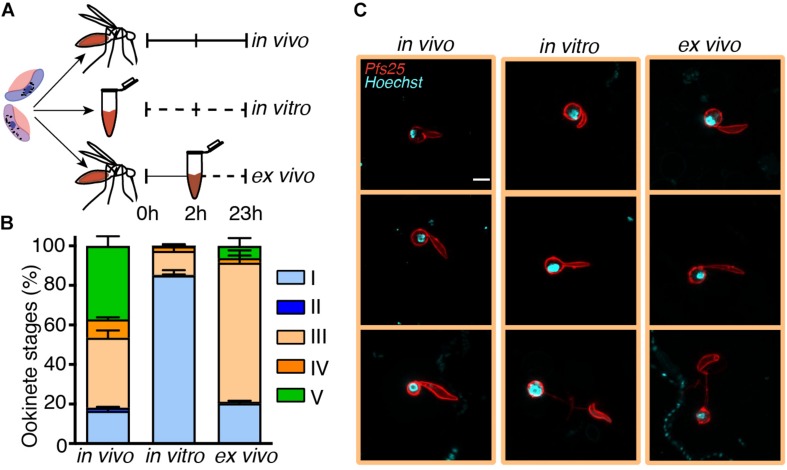
Ookinete development *in vitro* is critically impaired at the stage III-to-IV transition. The same *Plasmodium falciparum* gametocyte culture was used for mosquito infections or for *in vitro* ookinete development. To bypass the fertilization block, 20 mosquito blood boluses per time point were pooled and dissected 2 h post infection (hpi), and the residing parasites were transferred into *in vitro* cultures and incubated for 21 h (*ex vivo* conditions). Parasites developed in the mosquito bolus or in cultures *in vitro* were stained with anti-Pfs25 antibody at 2 and 23 hpi or post culture setup and were classified according to stages. **(A)** Schematic overview of the experiment. **(B)** Proportional composition of Pfs25-positive parasite stages developed *in vivo*, *in vitro*, and *ex vivo* 23 hpi or post culture as depicted in **(A)**. Bar plots represent the mean proportions ± SEM (*N* = 3, *n* = 200 parasites counted per time point). **(C)** Representative images of anti-Pfs25-positive and Hoechst-stained stage III ookinetes 23 hpi or post culture developed in three conditions (scale bar-5 μm). The *z*-test was used to perform pairwise comparison of mean proportions of each ookinete stage and their variances *in vivo*, *in vitro*, and *ex vivo*.

Statistical analyses for Figures 3, 4 were performed by comparing the fraction of each single ookinete stage between two growth conditions at the same time point. The method (see below) takes both the biological variability and the sample sizes into account and allows estimation of the errors associated with the average percentages, which are then compared using a *z*-test. We defined f1, f2, and f3 as the fractions of the morphology for three independent experiments under one growth condition and s1, s2, and s3 as the corresponding sample sizes. For each of the three sample sizes, we take one fraction at random and generate one number of ookinetes from a binomial distribution with probability given by the chosen fraction and population size given by the sample size. As an illustrative example, suppose that we associate f3 to s1, f1 to s2, and f1 to s3 for a certain ookinete stage (e.g., knob) at a certain time (e.g., 17 hpi) and a certain condition (e.g., *in vivo*). We then generate three random numbers from binomial distributions with the parameters given by the fraction and sample size. These three numbers (n1, n2, and n3) are the number of ookinetes found for the three samples. This corresponds to a simulated replica of the same experiment under the condition that the fractions (f1, f2, and f3) and the sample sizes (s1, s2, and s3) are the same as found in the experiment. The corresponding fraction of ookinete will then be g1 = n1/s1, g2 = n2/s2, and g3 = n3/s3.

We repeat this procedure *M* times, for *M* = 3e+6. After this procedure, the data contain *M* triplets of random numbers g1, g2, and g3 that represent simulated outcomes of the same experiments given the data obtained in the experimental work. The average of each of the triplets produces *M* average fractions *E*[g] = (g1 + g2 + g3)/3. The mean *E*[E(g)] of all these *M* average fractions and the variance Var[E(g)] of the *M* average fractions are the mean fraction and its variance associated with the ookinete stage at that given time point and under the given condition. We repeat this procedure for the each ookinete stage, time point, and condition and then compare the means of two conditions in pairs, taking the variances thus computed into account. The *z*-test applied to the two means and their variances finally delivers the *p*-value. Based on the above-mentioned procedure, the generated *p*-values are summarized in the Table 1 (refer to Figures 3C,D) and Table 2 (refer to Figure 4). The raw data used for this analysis are reported in Supplementary Tables 4–6.

**TABLE 1:** *p*-values computed from the *z*-test for the comparison *in vivo* versus *in vitro* as shown in Figures 3C,D.

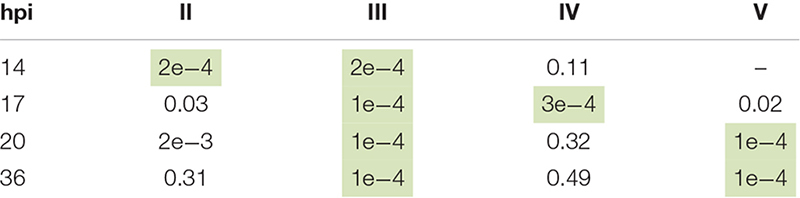

*Significant *p*-values (smaller than 0.001, in green) indicate the arrest of development at stage III *in vitro* compared to *in vivo*, where instead the ookinetes reach the mature stage at 20 h post infection (hpi). *p*-values smaller than the inverse of the product of the sample sizes have been replaced with 1e−4.*

**TABLE 2:** *p*-values computed with a *z*-test for the comparison *in vivo*, *in vitro*, and *ex vivo* at 23 h post infection (hpi) as shown in Figure 4B.

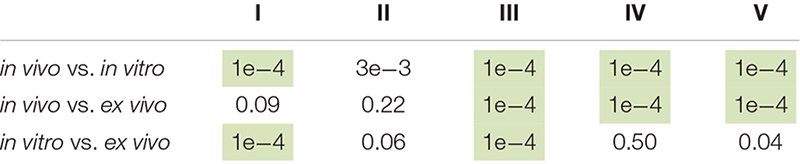

*Significant *p*-values (smaller than 0.001, in green) indicate that *in vitro* ookinetes are arrested at stage I, whereas *in vivo* and *ex vivo* cells have a similar stage I and II development. Both *in vitro* and *ex vivo* ookinetes have a further blockage point at stage III compared to the *in vivo* conditions. *p*-values smaller than 1e−4 have been replaced with 1e−4.*

## Results

### Stages of *P. falciparum* Ookinete Development in *A. coluzzii*

To characterize the major stages of zygote development, we performed a time course fluorescence live microscopy analysis of the blood bolus content of *A. coluzzii* mosquitoes infected with gametocyte cultures of *P. falciparum*. Live parasites within the mosquito bolus were dissected at 11, 14, 17, 20, 23, and 36 hpi and stained with the Hoechst nuclear dye and anti-Pfs25 antibodies. We identified five parasite developmental stages based on morphology, fluorescence patterns, and nuclear position (Figure 1A). Specifically, all immature stages were round shaped and displayed differences in the length of the protuberance that eventually gave the elongated shape to mature ookinetes. Stage I lacked the protuberance and was indistinguishable from egressed female gametes; stage II featured a short protuberance that did not exceed in length the radius of the main spherical body (knob); stage III had an elongated well-developed protuberance of at least the length of the radius of the main spherical body (stem); stage IV had a round nucleated body of reduced size and a wide long protuberance; stage V acquired the characteristic elongated shape with or without attached residual spherical body (Figure 1A). Importantly, stages I–IV displayed nuclear staining in the round body. Nuclear translocation from the residual round body to the well-formed elongated protuberance was observed during the transition from stage IV to stage V (Supplementary Figure 1). We also detected parasites that were suggestive of a degenerated form of a mature ookinete with disrupted Pfs25 surface signal and named them ghosts (Figure 1A). As these forms were only observed after appearance of mature ookinetes at the late time point of ookinete development *in vivo*, we speculate that ghosts represent ookinetes undergoing degradation probably due to the harsh environment of the mosquito midgut. This classification was used to measure the proportion of each stage in the mosquito blood bolus at six time points after infection. We found no obvious morphological changes in the round-shaped parasites until 11 hpi. During the next 3–14 h, the zygotes progressively developed protuberance, translocated the nucleus from the shrinking round body to the protuberance, and converted into mature ookinetes. We established the following kinetics of the ookinete development: stage II dominated at 14 hpi, stage III at 17 hpi, and stage IV between 17 and 23 hpi (Figures 1B,C and Supplementary Table 2). Mature ookinetes appeared at 17 hpi, peaked at 23 hpi, and persisted in the blood bolus until 36 hpi, whereas the ghost forms accumulated between 23 and 36 hpi (Figures 1B,C and Supplementary Table 2). The absolute number of Pfs25-positive parasites per mosquito blood bolus remained constant until 23 hpi but decreased at 36 hpi (Supplementary Figure 2 and Supplementary Table 2), likely reflecting the ookinete traversal of the mosquito midgut. Interestingly, only half of bolus-resident parasites developed into ookinetes, suggesting that at some stages, parasite development was restricted by the mosquito environment. We concluded that the observed progressive changes in proportions of developmental stages were not caused by parasite mortality but represented the dynamics of ookinete development in the mosquito.

### Development of Mature Ookinetes in the Mosquito Blood Bolus Reliably Predicts Successful Mosquito Infection

As only half of the ingested parasites transformed into mature ookinetes (Figure 1B), we next evaluated how well the exflagellation rate of a given gametocyte culture, the conversion rate of egressed gametes into all ookinete stages (stages II to V) or into mature ookinetes only (stage V), predicted the success of oocyst formation. Ookinete development was monitored by anti-Pfs25 staining at 24 hpi as described above, whereas oocysts were labeled by mercurochrome staining for counting at 11 days post infection (dpi). In line with the field studies, gametocyte exflagellation rate (accounting only for male gametocytes) was a poor predictor of oocyst prevalence (Figure 2A). We found that oocyst prevalence (number of oocyst-positive mosquitoes/total number of blood fed mosquitoes) correlated significantly with the ookinete conversion rate [number of converted ookinetes (stages II to V)/total Pfs25-positive parasites] and the proportion of mature ookinetes [number of mature ookinetes (stage V)/total Pfs25-positive parasites] (Figures 2B,C and Supplementary Table 3). We concluded that similar to natural infections, ookinete conversion is a more reliable predictor of parasite development in the mosquito than exflagellation rate, pointing to some critical steps that regulate gamete fusion and early stages of zygote development.

### Critical Stages of Ookinete Development *in vitro*

To identify developmental bottlenecks in ookinete conversion, we compared the dynamics of *in vivo* and *in vitro* ookinete development using the same initial gametocyte culture for mosquito infections and ookinete culture. At selected time points, parasites were isolated from the mosquito bolus or collected from the culture, stained with anti-Pfs25 antibodies and staged as described above. The absolute number of Pfs25-positive parasites stayed overall constant in all conditions until 23 hpi (Supplementary Figure 3). As expected, at 23 hpi, the majority of zygotes in the mosquito bolus developed protuberances, and 40% of them converted into mature ookinetes. In contrast, at the same time point *in vitro*, most of the detected Pfs25-positive forms remained at stage I (Figures 3A,B). As gametocytes efficiently egressed in both conditions (Supplementary Table 1), our findings are consistent with the previous reports that proposed a block in gamete fertilization *in vitro*. Focusing the analysis on the small proportion of ookinetes that developed from stage II onward, we observed an accumulation of stage III parasites. While this stage was dominant at the 14 h post culture setup, the corresponding samples *in vivo* showed a higher proportion of stage II parasites (Figures 3C,D, *p*-values = 0.0002, paired *z*-test). Interestingly, stage III parasites continued to accumulate in cultures *in vitro* at later time points, and only a small proportion of the parasites developed into mature ookinetes. In contrast, slower development of stage III forms *in vivo* was accompanied by swift progression to stage IV and V ookinetes. Our results identified a specific critical step in ookinete development after fertilization–the rate of stage III to stage IV transition–morphologically corresponding to a proper enlargement of the ookinete protuberance.

We next attempted to rescue *ex vivo* the critical step of ookinete development blocked *in vitro*. In these experiments, mosquitoes were infected with the gametocyte cultures, and 2 h later, the parasites were extracted from the insect gut and put into culture *in vitro* for an additional 21 h. In parallel, we examined the dynamics of ookinete development from the same gametocyte culture *in vivo* and *in vitro* as described above (Figure 4A). As the mosquito environment is not sterile, the *ex vivo* isolated parasites were treated with an antibiotic/antimycotic mix to prevent the overgrowth of the microbes inhabiting the mosquito midgut. This antimicrobial treatment had no effect on parasite development (Supplementary Figure 4). We found that the proportion of ookinetes that bypassed stage I was not significantly different in *in vivo* versus *ex vivo* conditions, demonstrating that the first 2 h of *in vivo* development rescued the fertilization block *in vitro* to the levels observed *in vivo* (Figure 4B, *p*-value = 0.09, paired *z*-test). However, the majority of the *ex vivo* parasites remained at stage III at 23 hpi, with only a minor fraction developing into mature ookinetes (*p-*value = 0.0001, paired *z*-test). The arrested stage III parasites featured a much thinner protuberance than those obtained *in vivo* (Figure 4C). Altogether, our results describe a new critical step in ookinete development. Further studies should investigate cellular mechanisms that control protuberance emergence and enlargement in *P. falciparum*.

## Discussion

The human-to-mosquito *Plasmodium* transition is essential for transmission of malaria parasites and a promising target of drug- or antibody-mediated transmission-blocking interventions. With both pharmacological and immunological interventions receiving increased attention, the absence of robust ookinete *in vitro* culturing methods poses a major roadblock for identification of potent inhibitory targets. Here, by comparing the dynamics of ookinete development *in vivo* and *in vitro*, we characterize mosquito-regulated critical steps in early ookinete maturation. The first mosquito factor shaping host-to-vector transmission was identified almost 40 years ago and was shown to increase exflagellation efficiency of male gametocytes in the avian malaria parasite *Plasmodium gallinaceum* (Nijhout, 1979). Later on, this factor was purified from mosquito extracts and identified as xanthurenic acid (Billker et al., 1998). The results reported here postulate an existence of additional mosquito factors that promote gamete fertilization and zygote morphogenesis.

We found that the rate of gamete conversion to ookinetes was a good predictor of oocyst production in the mosquito, whereas the rate of production of male gametes (exflagellation) was not. These observations indicate that the first 24 h are crucial for *Plasmodium* establishment in its vector. These results are in line with the field studies that showed that numbers of circulating gametocytes do not always predict parasite infectiousness to the insect vector (Bousema and Drakeley, 2011). A meta-analysis of the infectiousness of *P. falciparum*-infected blood samples from children from Burkina Faso and Tanzania to *A. gambiae*, and a similar study with *P. vivax* and *A. dirus*, revealed a non-linear relationship between the gametocyte titers in the donors’ blood and infection prevalence in the mosquito (Churcher et al., 2013; Kiattibutr et al., 2017). Furthermore, a recent meta-analysis conducted on *P. falciparum* gametocyte carriers in Mali, Burkina Faso, and Cameroon revealed a broad positive correlation between circulating female gametocytes and infected mosquitoes, while circulating male gametocytes contributed to mosquito infection only at low gametocyte densities (Bradley et al., 2018). These findings provide an additional proof that the *Plasmodium* exflagellation rate, which corresponds to the fraction of mature male gametocytes, is a poor predictor of carrier infectivity to mosquitoes.

We propose that the poor predictive properties of gamete activation are linked to variability in mosquito factors that control fertilization and zygote development. The importance of the mosquito environment is supported by two observations: (i) rescue of gamete fertilization block by within-mosquito development during the first 2 h after infection and (ii) abnormal morphogenesis of stage III parasites *in vitro* that results in narrow protuberance, which may block nuclear migration at stage IV. Interestingly, the defined here ordering of stages III and IV differs from that proposed by Ghosh et al. (2010), where the ookinetes with enlarged stem (stage IV here) were positioned before the forms with the elongated stem (stage III here). Therefore, the systematic time-series approach applied here contributed to a better understanding of the timing and order of the crucial steps in ookinete development.

The synchronous comparison of *in vivo* and *in vitro* ookinete maturation confirmed that *P. falciparum* gamete fertilization is very inefficient *in vitro* (Delves et al., 2017) (Graphical Abstract). Here, we demonstrate that this block is completely reverted by allowing *in vivo* parasite within-mosquito development within the first 2 h. Our cultures *in vitro* contained xanthurenic acid, and we detected no differences in gamete egress between *in vivo* and *in vitro* conditions. Therefore, we propose that gamete fusion requires other mosquito factor(s). Currently, the nature of these factors is unknown. Interestingly, our conclusions are in line with an earlier study which used a computational approach to model ookinete development (Lawniczak and Eckhoff, 2016).

In contrast to fertilization, the short pulse of *in vivo* development did not rescue the second block in the ookinete maturation *in vitro*. Instead of delay, we observed faster development of the protuberance and, hence, accumulation of stage III *in vitro*. However, this faster development translated into morphological abnormalities, as stage III developed thinner protuberance *in vitro*. This observation could be explained by higher concentrations of some permissive factor(s) in the culture medium as compared to the natural mosquito environment. Identification of the factor(s) not only will allow optimization of *in vitro* conditions but also will inform how within-mosquito variability in this factor affects ookinete development *in vivo*. In conclusion, the results presented here suggest that successful *P. falciparum* fertilization and ookinete maturation require the continuous presence of multiple as yet unknown mosquito factors. Identification of these factors should benefit the establishment of *P. falciparum* ookinete culture *in vitro* and uncover the mechanisms that regulate early stages of transmission of this deadly parasite.

## Data Availability Statement

All datasets generated for this study are included in the articleSupplementary Material.

## Author Contributions

GS, GC, and PS-C conceived and performed the experiments, analyzed the results, and wrote the manuscript. AV performed statistical analyses and wrote the manuscript. PA and EL conceived experiments, analyzed the data, and wrote the manuscript.

## Conflict of Interest

The authors declare that the research was conducted in the absence of any commercial or financial relationships that could be construed as a potential conflict of interest.
