# Supplementary material for: Critical Steps of Plasmodium falciparum Ookinete Maturation
Source: Front Microbiol. 2020 Mar 17;11:269. doi: 10.3389/fmicb.2020.00269 (PMC7092720; doi:10.3389/fmicb.2020.00269)
Supplement: Supplementary file 1 [file Data_Sheet_1.docx]

## Supplementary Figures

**
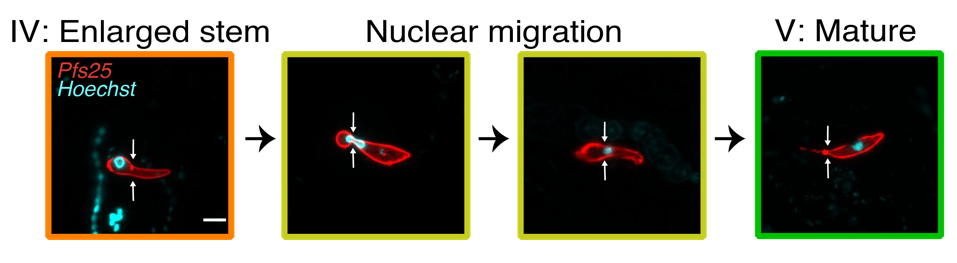
**

**Supplementary Figure 1. Nuclear migration during the transition from stage IV to stage V ookinetes**

*A. coluzzii* mosquitoes were infected with *P. falciparum* gametocytes. Live parasites were isolated from the mosquito bolus 23 h post infection and stained with anti-Pfs25 antibody. Representative images of stage IV, stage V and intermediate ookinete stages showing nuclear migration. White arrows indicate the connection between the body and the protuberance, through which nucleus migrates. Scale bar - 5 µm.

**
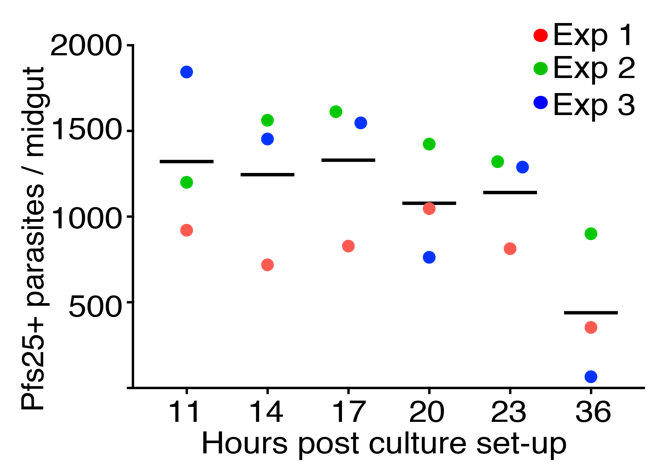
**

**Supplementary Figure 2. Quantification of Pfs25-positive ookinetes in *in vivo* time-series experiments (**refers to **Figure 1).**

Absolute number of Pfs25-positive (Pfs25+) parasites per midgut were counted in the experiments shown in **Figure 1A**. Horizontal lines show means of independent color-coded experiments (N = 3).

**Supplementary Figure 3. In parallel quantification of Pfs25-positive ookinetes in *in vivo* and *in vitro* time-series experiments.**

Absolute numbers of Pfs25-positive (Pfs25+) parasites per gut (*in vivo*) or per ml of culture medium (*in vitro*) were counted at the indicated time points after infection in experiments shown in **Figure 3A**. Every point represents the mean of independent experiments (N = 3) (**Figure 3**: n = 200 in **A,C**; and n = 500 in **B,D**).

# Supplementary Figure 4. Gentamycin treatment does not affect ookinete conversion *in vitro.*

In order to exclude a detrimental effect of the antibiotic/antimycotic treatment in *ex vivo* experiments (see also **Figure 4**)*, in vitro* cultures subjected to Fungizone treatment (2 min) 2 h after gametocytogenesis induction, washed and resuspended in complete medium supplemented with 40 μg/ml Gentamicin (+) were compared with *in vitro* cultures without Gentamycin (-). Both conditions were staged for ookinete development as in **Figure 2A** after 23 h of incubation. Percentage of ookinete stages out of total Pfs25-positive parasites is indicated. Bar plots represent the mean proportions ± SEM of independent experiments (N = 3; n = 200).

|  | ***in vivo*** |
| --- | --- |
| Gametocytes ingested /midgut | 7.4 x 10^3^ |
| Pfs25+/midgut (mean) | 1.5 x 10^3^ |
| Egressed parasites in the bolus (%) | 20 |

# Supplementary Tables

|  | ***in vitro*** |
| --- | --- |
| Gametocytes /ml | 5.0 x 10^6^ |
| Pfs25+/ml (mean) | 1.5 x 10^6^ |
| Egressed parasites (%) | 30 |

**Supplementary Table 1. Gamete egress rate *in vitro* and *in vivo*.**

Gamete egress rate in *in vivo* or *in vitro* cultures conditions was estimated by calculating the proportion of egressed Pfs25-positive (Pfs25+) parasites among all mature gametocytes. For the *in vivo* condition, the number of mature gametocytes ingested/midgut was calculated by multiplying the final concentration of mature gametocytes in the feeder (3.7 x 10^3^ gametocyte/µl) by 2 μl of ingested blood meal per mosquito. For the *in vitro* conditions, mature gametocytes were diluted in the initial culture to 5 x 10^6^/ml as indicated in Material and Methods. The number of Pfs25+ (egressed) parasites was enumerated in *in vivo* (per midgut) and *in vitro* (per ml of culture) using a hemocytometer, and was not significantly different at the tested time points (8, 14, 17 and 20 h post infection or post culture, see **Figure S3**). Therefore, we estimated the number of Pfs25+ parasites in the two conditions as a mean of the values obtained at different time points indicated above. Raw data are reported in **Supplementary Tables 4** and **5** (N = 3).

| Stage | I | | | II | | | III | | | IV | | | IV | | | Ghost | | | Pfs25+/midgut | | |
| --- | --- | --- | --- | --- | --- | --- | --- | --- | --- | --- | --- | --- | --- | --- | --- | --- | --- | --- | --- | --- | --- |
| Exp. | 1 | 2 | 3 | 1 | 2 | 3 | 1 | 2 | 3 | 1 | 2 | 3 | 1 | 2 | 3 | 1 | 2 | 3 | 1 | 2 | 3 |
| 11 hpi | 141 | 181 | 166 | 58 | 18 | 31 | 1 | 1 | 3 | 0 | 0 | 0 | 0 | 0 | 0 | 0 | 0 | 0 | 921 | 1200 | 1844 |
| 14 hpi | 84 | 76 | 99 | 66 | 36 | 40 | 50 | 88 | 60 | 0 | 0 | 1 | 0 | 0 | 0 | 0 | 0 | 0 | 718.8 | 1563 | 1453 |
| 17 hpi | 48 | 36 | 65 | 31 | 16 | 11 | 106 | 96 | 109 | 10 | 23 | 15 | 5 | 29 | 0 | 0 | 0 | 0 | 828.1 | 1613 | 1547 |
| 20 hpi | 23 | 21 | 40 | 9 | 9 | 14 | 48 | 61 | 75 | 21 | 16 | 18 | 99 | 93 | 53 | 0 | 0 | 0 | 1047 | 1423 | 762.6 |
| 23 hpi | 15 | 14 | 41 | 5 | 4 | 5 | 53 | 48 | 61 | 16 | 20 | 28 | 111 | 114 | 65 | 0 | 0 | 0 | 812.5 | 1321 | 1289 |
| 36 hpi | 1 | 3 | 37 | 5 | 0 | 0 | 13 | 14 | 11 | 14 | 5 | 4 | 107 | 138 | 26 | 60 | 40 | 122 | 353.1 | 900 | 65.6 |

**Supplementary Table 2**. **Kinetics of parasite maturation (**refers to **Figure 1** and **Figure S2).**

The numbers of parasites for each stage (n = 200) and total numbers of Pfs25-positive (Pfs25+) parasites per midgut at each time point (h post infection, hpi) are indicated (N = 3).

| Exp. | Exflagellation (%) | I | II+III+IV | V | Oocyst prevalence (%) |
| --- | --- | --- | --- | --- | --- |
| 1 | 29.0 | 187 | 12 | 1 | 3.3 |
| 2 | 21.2 | 23 | 93 | 84 | 90.0 |
| 3 | 41.0 | 29 | 85 | 86 | 66.6 |
| 4 | 29.0 | 71 | 79 | 50 | 46.6 |
| 5 | 26.5 | 48 | 96 | 56 | 50.0 |
| 6 | 18.7 | 70 | 89 | 41 | 46.6 |
| 7 | 46.0 | 15 | 85 | 100 | 96.6 |
| 8 | 14.0 | 39 | 85 | 76 | 63.3 |
| 9 | 32.0 | 25 | 91 | 84 | 73.3 |
| 10 | 30.0 | 16 | 62 | 122 | 40.0 |
| 11 | 40.0 | 8 | 41 | 151 | 96.6 |
| 12 | 22.2 | 36 | 75 | 89 | 76.6 |
| 13 | 26.8 | 26 | 60 | 114 | 86.6 |
| 14 | 22.0 | 21 | 32 | 147 | 100.0 |
| 15 | 24.5 | 24 | 108 | 68 | 80.0 |
| 16 | 19.3 | 38 | 130 | 32 | 60.0 |
| 17 | 33.4 | 18 | 76 | 106 | 76.6 |

**Supplementary Table 3**. **Exflagellation, ookinete conversion and oocyst prevalence data (**refers to **Figure 2).**

Exflagellation rate, number of each ookinete stage at 24 h post infection (n = 200) and oocyst prevalence in mosquitoes (n = 30) 11 days post infection are indicated for each experiment (N = 17).

| Stage | I | | | II | | | III | | | IV | | | V | | | Ghost | | | Pfs25+/midgut | | |
| --- | --- | --- | --- | --- | --- | --- | --- | --- | --- | --- | --- | --- | --- | --- | --- | --- | --- | --- | --- | --- | --- |
| Exp. | 1 | 2 | 3 | 1 | 2 | 3 | 1 | 2 | 3 | 1 | 2 | 3 | 1 | 2 | 3 | 1 | 2 | 3 | 1 | 2 | 3 |
| 8 hpi | 200 | 200 | 200 | 0 | 0 | 0 | 0 | 0 | 0 | 0 | 0 | 0 | 0 | 0 | 0 | 0 | 0 | 0 | 726 | 1187 | 625 |
| 14 hpi | 99 | 74 | 118 | 40 | 46 | 33 | 60 | 79 | 49 | 1 | 1 | 0 | 0 | 0 | 0 | 0 | 0 | 0 | 1453 | 1078 | 2625 |
| 17 hpi | 65 | 34 | 62 | 11 | 9 | 15 | 109 | 104 | 90 | 15 | 37 | 17 | 0 | 16 | 16 | 0 | 0 | 0 | 1547 | 1390 | 2219 |
| 20 hpi | 40 | 15 | 30 | 14 | 5 | 7 | 75 | 52 | 82 | 18 | 39 | 23 | 53 | 89 | 58 | 0 | 0 | 0 | 763 | 1328 | 2844 |
| 36 hpi | 37 | 11 | 5 | 0 | 0 | 2 | 11 | 15 | 28 | 4 | 6 | 37 | 26 | 92 | 77 | 122 | 76 | 51 | 66 | 378 | 781 |

**Supplementary Table 4. Maturation of ookinetes *in vivo* (**refers to **Figure 3A,C** and **Figure S3).**

The number of parasites at each stage (n = 200) and total number of Pfs25-positive (Pfs25+) parasites per midgut at each time point (h post infection, hpi) are indicated (N = 3).

| Stage | I | | | II | | | III | | | IV | | | V | | | Ghost | | | Pfs25+/ml | | |
| --- | --- | --- | --- | --- | --- | --- | --- | --- | --- | --- | --- | --- | --- | --- | --- | --- | --- | --- | --- | --- | --- |
| Exp. | 1 | 2 | 3 | 1 | 2 | 3 | 1 | 2 | 3 | 1 | 2 | 3 | 1 | 2 | 3 | 1 | 2 | 3 | 1 | 2 | 3 |
| 8 hpi | 500 | 500 | 500 | 0 | 0 | 0 | 0 | 0 | 0 | 0 | 0 | 0 | 0 | 0 | 0 | 0 | 0 | 0 | 910930 | 2463000 | 1257200 |
| 14 hpi | 490 | 444 | 475 | 0 | 10 | 5 | 10 | 46 | 20 | 0 | 0 | 0 | 0 | 0 | 0 | 0 | 0 | 0 | 1621900 | 850000 | 1117000 |
| 17 hpi | 482 | 462 | 480 | 1 | 0 | 0 | 17 | 38 | 19 | 0 | 0 | 1 | 0 | 0 | 0 | 0 | 0 | 0 | 1801500 | 1743700 | 2151000 |
| 20 hpi | 486 | 458 | 442 | 0 | 0 | 0 | 11 | 31 | 43 | 2 | 8 | 12 | 1 | 3 | 3 | 0 | 0 | 0 | 1525000 | 1343700 | 1267200 |
| 36 hpi | 477 | 479 | 481 | 1 | 0 | 0 | 14 | 15 | 18 | 5 | 4 | 0 | 3 | 2 | 1 | 0 | 0 | 0 | 1365600 | 1807800 | 1306200 |

**Supplementary Table 5. Maturation of ookinetes *in vitro* (**refers to **Figure 3B,D** and **Figure S3).**

The number of parasites for each stage (n = 200) and total number of Pfs25-positive (Pfs25+) parasites per ml of culture at each time point (h post culture, hpi) are indicated (N = 3).

| Stage | I | | | II | | | III | | | IV | | | V | | | Ghost | | |
| --- | --- | --- | --- | --- | --- | --- | --- | --- | --- | --- | --- | --- | --- | --- | --- | --- | --- | --- |
| Exp. | 1 | 2 | 3 | 1 | 2 | 3 | 1 | 2 | 3 | 1 | 2 | 3 | 1 | 2 | 3 | 1 | 2 | 3 |
| *In vivo* | 37 | 25 | 37 | 4 | 2 | 4 | 85 | 67 | 60 | 19 | 22 | 15 | 55 | 84 | 84 | 0 | 0 | 0 |
| *In vitro* | 162 | 180 | 168 | 1 | 0 | 0 | 35 | 15 | 23 | 2 | 3 | 9 | 0 | 2 | 0 | 0 | 0 | 0 |
| *Ex vivo* | 45 | 36 | 41 | 3 | 0 | 2 | 118 | 161 | 143 | 6 | 0 | 8 | 28 | 3 | 6 | 0 | 0 | 0 |

**Supplementary Table 6. Maturation of ookinetes *ex vivo* (**refers to **Figure 4).**

The number of parasites at each stage (n = 200) at each time point are indicated (N = 3).
